# Genome-Wide Characterization and Sequence Polymorphism Analyses of *Glycine max* Fibrillin (*FBN*) Revealed Its Role in Response to Drought Condition

**DOI:** 10.3390/genes14061188

**Published:** 2023-05-29

**Authors:** Muhammad Zeshan Zafer, Muhammad Hammad Nadeem Tahir, Zulqurnain Khan, Muhammad Sajjad, Xiangkuo Gao, Muhammad Amir Bakhtavar, Ummara Waheed, Maria Siddique, Zhide Geng, Shoaib Ur Rehman

**Affiliations:** 1 SINO-PAK Joint Research Laboratory, Institute of Plant Breeding and Biotechnology, Muhammad Nawaz Shareef, University of Agriculture, Multan 66000, Pakistan; ranazeeshannoon347@gmail.com (M.Z.Z.); zulqurnain.khan@mnsuam.edu.pk (Z.K.); 2 Institute of Plant Breeding and Biotechnology, Muhammad Nawaz Shareef, University of Agriculture, Multan 66000, Pakistan; hammad.nadeem@mnsuam.edu.pk (M.H.N.T.); amir.bakhtavar@mnsuam.edu.pk (M.A.B.); ummara.waheed@mnsuam.edu.pk (U.W.); 3 Department of Biosciences, COMSATS University, Islamabad (CUI), Park Road, Islamabad 45550, Pakistan; muhammad.sajjad@comsats.edu.pk; 4 Institute of Food Crops, Yunnan Academy of Agricultural Sciences, Kunming 650204, China; 5 Department of Environmental Sciences, COMSATS University Islamabad, Abbottabad Campus, Abbottabad 22060, Pakistan; maria@cuiatd.edu.pk

**Keywords:** *Glycine max*, fibrillin (*FBN*), genome-wide identification, marker-assisted breeding, CAPS, drought

## Abstract

The fibrillin (*FBN*) gene family is widely distributed in all photosynthetic organisms. Members of this gene family are involved in plant growth and development and their response to various biotic and abiotic stress factors. In this study, 16 members of *FBN* were identified in *Glycine max* and characterized by using different bioinformatics tools. Phylogenetic analysis classified *FBN* genes into seven groups. The presence of stress-related *cis*-elements in the upstream region of *GmFBN* highlighted their role in tolerance against abiotic stresses. To further decipher the function, physiochemical properties, conserved motifs, chromosomal localization, subcellular localization, and *cis*-acting regulatory elements were also analyzed. Gene expression analysis based on FPKM values revealed that *GmFBNs* greatly enhanced soybean drought tolerance and controlled the expression of several genes involved in drought response, except for *GmFBN-4*, *GmFBN-5*, *GmFBN-6*, *GmFBN-7* and *GmFBN-9*. For high throughput genotyping, an SNP-based CAPS marker was also developed for the *GmFBN-15* gene. The CAPS marker differentiated soybean genotypes based on the presence of either the *GmFBN-15-G* or *GmFBN-15-A* alleles in the CDS region. Association analysis showed that *G. max* accessions containing the *GmFBN-15-A* allele at the respective locus showed higher thousand seed weight compared to accessions containing the *GmFBN-15-G* allele. This research has provided the basic information to further decipher the function of *FBN* in soybean.

## 1. Introduction

Soybean (*Glycine max* L.) is an important source of edible oil. Like other crops, abiotic stress factors are major limiting factors affecting its yield and quality. Hence, finding and understanding the role of stress tolerance-causing genes in soybean is an important step toward soybean breeding [1,2]. The availability of soybean PAN-genome has also opened up new insights for molecular breeding in soybean [3]. Soybean PAN-genome is also being used to explore sequence polymorphism within genome and to convert these polymorphic sites into breeding tool kits in the shape of SNP-based molecular markers [1,2].

The genome-wide identification and characterization of plant transcription factors (TFs) bears key importance. The TFs play a major role in plant growth and developmental processes, including tolerance against biotic and abiotic stress factors. Lipid-associated proteins, fibrillin (FBN), are mainly present in plastids and are omnipresent in plants. Fibrillin was first found in fibrils in the chromosomes of dog rose (*Rosa canina*) and bell pepper (*Capsicum annuum*) [4,5]. Since then, several organelles, such as chloroplast plastoglobulus (PGs) and algal eyespots, have been shown to contain FBN proteins. FBN members are known by a broad variety of names, such as plastoglobuli (PGL), plastid-lipid-associated protein (PAP), and chromoplast-specific carotenoid-associated protein (CaRP) [6,7]. Chlorophyll from cyanobacteria from some higher plants all include FBNs, which are essential for photosynthesis [8]. FBNs are a class of proteins essential for chloroplast development, lipid metabolism, and the evasion of stress in plants. Thylakoid stability and response to various stress stimuli have been linked to ***FBN* genes**. FBN proteins play a crucial function in photosynthesis and are involved in photoprotection in response to high-light conditions at low temperatures in cucumber [9]. The FBN protein family is composed of 12 sub-families. These sub-families have been identified in a wide range of organisms, i.e., from algae to higher plants, including wheat, Arabidopsis, tomato, and rice [10,11]. In wheat, besides being expressed in different tissues and developmental stages, *TaFBNs* are also expressed in response to biotic (stripe rust) and abiotic (drought, cold and heat) stresses [10]. Some *CaFBN* genes also upregulate in response to dehydration stress [12]. Kim et al. also reported the important role of *FBN* genes in plant growth and development [4]. *FBN* genes also contain many light-responsive elements and can promote plant height and flowering under high light levels in tobacco [13]. To date, *FBNs* have been characterized in a limited number of plant species, although their role in soybean molecular breeding for drought tolerance is yet to be reported.

To facilitate the use of *GmFBN* in soybean molecular breeding, the current study focused on the characterization of *GmFBN* for sub-cellular localization, conserved domain database (CDD), motif composition, *FBN* comparison among the species using sequence logo, comparative phylogenetic, molecular evolutionary analysis, and its potential roles in plant evolution followed by SNP-based marker development. Our findings outline that the *GmFBN* gene family offers a theoretical and practical foundation for analyzing their function, particularly in the context of soybean molecular breeding.

## 2. Materials and Methods

### 2.1. Sequence Identification

*AtFBNs* were downloaded from the TAIR database (https://www.arabidopsis.org/ accessed on 13 September 2022) and were used as reference. Pfam tools (http://pfam.xfam.org/ accessed on 15 September 2022) were used to identify the specific domain. This domain was BLAST on Phytozome (https://phytozome-next.jgi.doe.gov/ accessed on 21 September 2022) to acquire the putative genomic and protein sequences in targeted species. The soybean genome sequence and the general feature format file (GFF3) were downloaded from the online web server. Chemical properties were predicted by using the online web tool ExPASy ProtParam [14] (http://us.expasy.org/tools/protparam.html accessed on 11 October 2022). CELLO v.2.5 subcellular localization predictor (http://cello.life.nctu.edu.tw/ accessed on 21 October 2022) [15], Softberry (http://www.softberry.com/ accessed on 21 October 2022), and WoLF PSORT [16] (https://wolfpsort.hgc.jp/ accessed on 21 October 2022) were used to predict subcellular localization for *GmFBNs*. The total numbers of conserved domains in each *GmFBN* gene were determined using the NCBI CDD tool (https://www.ncbi.nlm.nih.gov/Structure/cdd/wrpsb.cgi accessed on 21 September 2022) and TBtools.

### 2.2. Phylogenetic Analysis of FBN

The protein sequences of *G. max*, *Arabidopsis thaliana*, *Glycine soja*, *Brachypodium distachyon*, *Gossypium hirsutum*, *Solanum. melongena*, *Populus. trichocarpa*, *Physcomitrella. patens*, *Zea mays*, and *Brassica. rapa* were aligned. MEGA11 was used to perform phylogenetic analysis using FBN protein sequences of the aforementioned species to determine the evolutionary history of FBN genes. Sequences were aligned using ClustalW, the while Neighbor-Joining (NJ) method was used to deduce evolutionary history [17]. To test the phylogenetic tree, the bootstrap method with 1000 replicates was used.

### 2.3. Gene Structure, Protein Motif and Cis-Acting Element Analysis

The GSDS 2.0.8. (http://gsds.gao-lab.org accessed on 7 October 2022) server was used to evaluate retrieved coding and genomic sequences from databases to identify the exon-intron structure. To examine the exon-intron structure, BED file and NJ phylogenetic tree data were used. The MEME web portal was utilized to analyze protein motif distributions. A PlantCARE database (accessed on 11 November 2022) [18] was to evaluate 2 kb upstream regions to characterize *cis*-elements based on their projected biological activities, and results were visualized using TBtools.

### 2.4. In-Silico Prediction and Physiochemical Properties of FBN in G. max

Physicochemical properties were predicted by ExPASy ProtParam (http://web.expasy.org/protparam/ accessed on 11 October 2022). Data regarding protein length, amino acid composition, protein molecular weight, *p*-value, and GRAVY score were retrieved for all *GmFBNs* using ExPASy ProtParam.

### 2.5. Sequence Logos of FBN Genes

To construct the sequence logos for conserved amino acid residues, multiple sequence alignment of *G. max*, *Z. mays*, and *A. thaliana* was carried out individually by Clustal X 2.0. (http://www.clustal.org/clustal2/ accessed on 16 November 2022), and sequence logos were created using WEBLOG (https://weblogo.berkeley.edu/logo.cgi accessed on 16 November 2022) [19].

### 2.6. Chromosomal Distribution and Synteny analyses of GmFBN

Soybean genome assemblies files were used to create a chromosomal length file, and data extracted from a physicochemical properties file were used to create a gene location file. These files were then visualized in PhenoGram (http://visualization.ritchielab.org/phenograms/plot accessed on 17 November 2022), following Kumar et al. [20]. To identify potential paralogous *FBN* gene pairings, a phylogenetic tree was used for interpretation. Rates of synonymous and non-synonymous substitution were calculated using TBtools on the resulting groups of two pairs. Additionally, the *Ka*/*Ks* ratio was also examined to determine the pattern of codon selection that occurred during evolution [21]. To perform the synteny analysis, Fa and GFF files of *A. thaliana* and *G. max* were used to generate the collinearity data. From previously generated data, collinearity, GFF, and CTL files were used for synteny visualization, following Yang et al. [22].

### 2.7. In-Silico Gene Expression Analysis of GmFBN

The transcript level values were retrieved from the NCBI GEO Dataset (Accession Number: GSE98958) and used to study the expression of *GmFBN* genes in dehydration and control conditions (https://www.ncbi.nlm.nih.gov/DataSets/ accessed on 12 December 2022). The *FBN* expression values (based on FPKM) were extracted to generate the heat map of *GmFBNs* expressed in response to drought. The heat map was constructed using TBtools software to express the transcript level of the *GmFBN* genes.

### 2.8. Soybean Plant Material and Phenotyping

Forty-six soybeans accessions were planted in 2021 at the MNS University of Agriculture, Multan. Following augmented design, the field experiment was conducted under both water-limited (WL) and well-water (WW) experimental units. The field was prepared by laser leveling followed by standard agronomic practices. Briefly, one bag of urea and di-ammonium phosphate per acre were applied before sowing. Each soybean genotype was planted (using 2–3 seed planting sites) in two beds, each measuring 15 feet long and 2.5 feet wide. Plant-to-plant spacing was strictly controlled at 1 foot. Plant thinning was also performed to eradicate unhealthy plants. Weeds were also controlled manually. The WL experimental units were subjected to drought conditions, specifically during the flowering period, while WW experimental units received irrigation once every two weeks. Data regarding maximum and minimum temperature, relative humidity, and rainfall is presented in Supplementary Table S1. Plant height, pods per plant, thousand seed weight, seed weight per pod, seed thickness, seeds per pod, seed length, seed width, and pod length were measured across both experimental units. Plant height was recorded at physiological maturity. Seed length, width, and thickness were measured using scanner S500A3B (Shenzhen Eloam Technology, China) from both water regimes [23]. Thousand seed weight was measured by weighing duplicate samples of almost 500 seeds from each soybean genotype [23].

### 2.9. Development of Single Nucleotide Polymorphism Based Cleaved Amplified Polymorphic Sequence (CAPS) Marker for GmFBN-15

The CTAB method was used to isolate genomic DNA from soybean seedling leaves [24]. DNA concentration was measured with a NANO-Drop (K5800C MicroSpectrophotometer) followed by running on 1.0% agarose gel to perform quality checks. After extraction, concentrated and diluted (100 ng/µL) DNA samples were placed at −80 °C for later usage.

The whole genome sequence of three cultivars of soybean (Williams-82, Fiskeby, and Lee) was downloaded from (https://phytozome-next.jgi.doe.gov/ 21 September 2022). The *GmFBN* sequences in the above-mentioned soybean cultivars were retrieved using Local BLAST. The Seqman program from the DNASTAR Lasergene package was used for multiple sequence alignment, which revealed sequence polymorphism only in *GmFBN-15*. This approach has been reported earlier by Nisar et al. and Fatima et al. [1,2]. Standard cleaved amplified polymorphic sequence (CAPS) guidelines were followed to develop the CAPS marker on the identified single nucleotide polymorphism (SNP) of *GmFBN-15.*

The CAPS marker was developed on the identified SNP of *GmFBN-15*. From the PAN-genome, we identified that Williams-82 carried the *GmFBN-15-A* allele at 433 nt, while Fiskeby and Lee contained the *GmFBN-15-G* at the respective location (https://www.soybase.org/dlpages/ accessed on 21 September 2022). Specific primers were designed flanking the SNP at 433 bp. The PCR recipe, 2.0 µL of genomic DNA at 100 ng/µL, 0.5 µL of forward and reverse primers at 10 µm, 6.0 µL of 2× PCR Master Mix (NOVOPROTIEN, China), and 4 µL ddH_2_O, was used to make a final volume of 13.0 µL. DNA thermal cycler (BIO-RAD, Model No. T100TM Thermal Cycler) was used to amplify the target region. The PCR reaction was: denaturation at 94 °C for 3 min, followed by multiple cycles (32 cycles for *GmFBN-15*) of denaturation at 94 °C for 30 s, annealing at temperature 56.7 °C for 30 s, extension at 72 °C for 25 s, and a 5 min final extension at 72 °C. The amplified PCR product was then subjected to digestion using *Bpi*I restriction enzyme. The conditions for enzymatic digestion were: 5.0 µL of PCR product, 0.2 µL *Bpi*I FastDigest enzyme, 1 µL 10× FastDigest Green Buffer, and 9.0 µL nuclease-free water for each sample, and incubation at 37 °C for ~20 min followed by gel electrophoresis. Agarose gel (2.5%) was used to separate the digested fragments, in 1× Tris-borate-EDTA (TBE) at 90 volts for 30 min.

### 2.10. Statistical Analyses

Descriptive statistics were conducted using Microsoft Excel 2019. The effect of each allelic variation on the observed phenotypic traits was analyzed using the Student’s *t*-test at a significance level of *p* < 0.05.

## 3. Results

### 3.1. Identification of FBNs Gene Family Members in Different Species

A total of 113 FBN genes were retrieved from nine different species, including *A. comosus* (angiosperm), *A. thaliana*, *G. max*, *G. soja*, *G. hirsutum*, *B. rapa* and *P. trichocarpa* (dicotyledons), *B. distachyon* and *Z. mays* (monocotyledons), and *P. patens* (Bryophyte). Among these, 16 FBN genes were identified in *G. max*, 16 in *G. soja*, 14 in *A. thaliana*, 11 in *Z. mays*, 12 in *B. rapa*, 14 in *G. hirsutum*, 11 in *P. trichocarpa*, 9 in *B. distachyon*, and 10 in *P. patens*. Gene IDs of the studied species are presented in Supplementary Table S2. We found that almost all the selected organisms had at least nine FBN genes (*B. distachyon* lowest number of FBNs), indicating that the FBN genes were subjected to large-scale expansion.

### 3.2. Conserved Domain, Gene Structure, Protein Motif Analysis and Cis-Acting Elements

**Figure 1:**
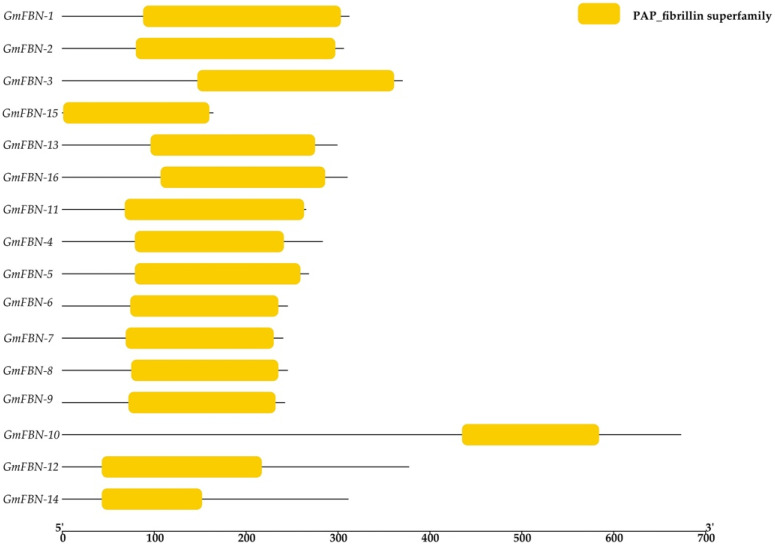
**GmFBN** conserved domain proteins. All GmFBN genes contained PAP superfamily domains. The amino acid length can be inferred by the ruler at the bottom.

**Figure 2:**
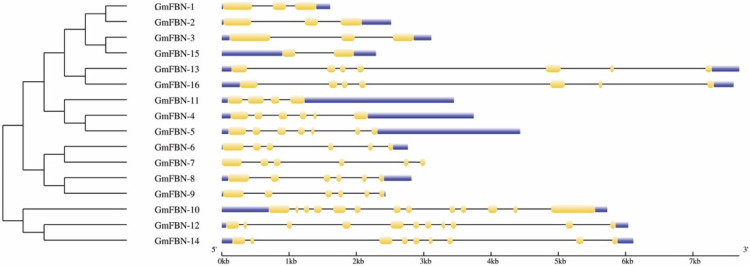
Gene structure analysis of GmFBN based on phylogenetic relationships. Yellow boxes indicate exon, blue boxes indicate the upstream and downstream regions, and black lines indicate intron.

**Figure 3:**
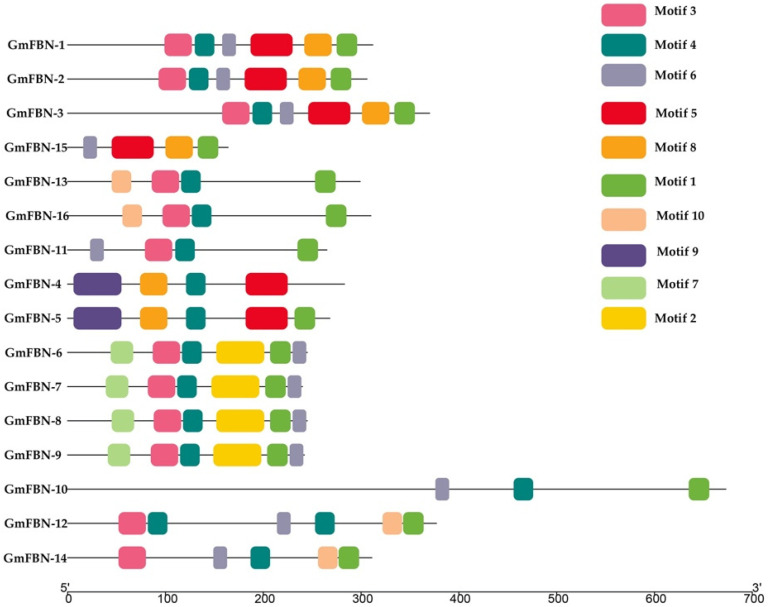
Conserved motifs prediction of GmFBN family. A Total of 10 motifs are presented in different colors. The amino acid length can be inferred by the ruler at the bottom.

**Figure 4:**
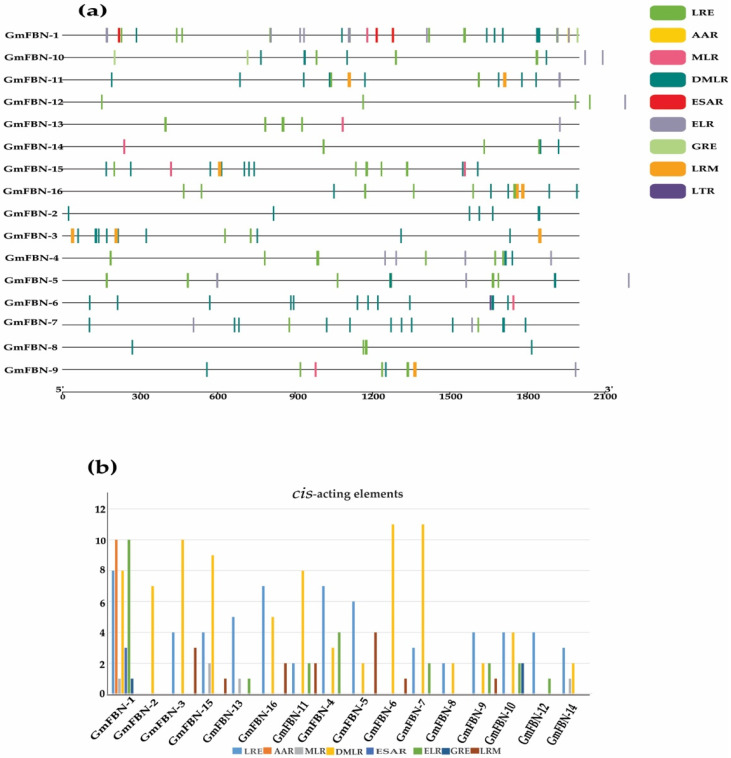
Composition of *cis*-acting regulatory elements in the promoters of GmFBNs. (**a**) Stress-responsive related elements, plant growth, and light-responsive related elements are shown by different colors represented by TBtools; (**b**) Graphical representation of *cis*-acting elements. LRE, light-responsive elements; AAR, amino acid responsive; MLR, methylene jasmonate responsive; DMLR, DNA module involved in light responsiveness; ESAR, elements involved in salicylic acid responsiveness; ELR, Ethylene responsive; GRE, gibberellin responsive; LTR, low temperature responsive; LRM, light responsive module.

Conserved motifs play a vital role in the basic functions of gene families. Multiple alignments of FBN protein sequences revealed that the FBN genes contained conserved the PAP fibrillin superfamily domain in all GmFBN genes. The PAP fibrillin superfamily domain was around 100 amino acids long and was present in the GmFBN genes (Figure 1). The structure of the PAP fibrillin domain, which is also a conserved domain, serves as a foundation for the categorization of fibrillin protein. The genomic and coding sequences were used to construct the gene structure. The intron numbers ranged from 1–11 (Figure 2). Using MEME, (Multiple Em for Motif Elicitation), we found that 16 GmFBN genes shared a total of 10 conserved motifs. There were a total of 6 motifs in GmFBN-1, GmFBN-2, and GmFBN-3. There were 4 and 5 in GmGBN-4 and GmFBN-5, respectively. Six each were present in GmFBN-6, GmFBN-7, GmFBN-8, and GmFBN-9. GmFBN-10 had 3, GmFBN-11 had 4, GmFBN-12 had 6, GmFBN-13 had 4, GmFBN-14 had 5, GmFBN-15, and GmFBN-16 each had 4 (Figure 3). Additionally, we identified *cis*-acting elements that were found in the upstream regions of GmFBN (Figure 4a). The *cis*-acting elements present in all GmFBNs were involved in processes such as plant growth, stress tolerance, and photosynthesis (Figure 4b).

### 3.3. Phylogenetic Analysis of FBNs Gene Family

**Figure 5:**
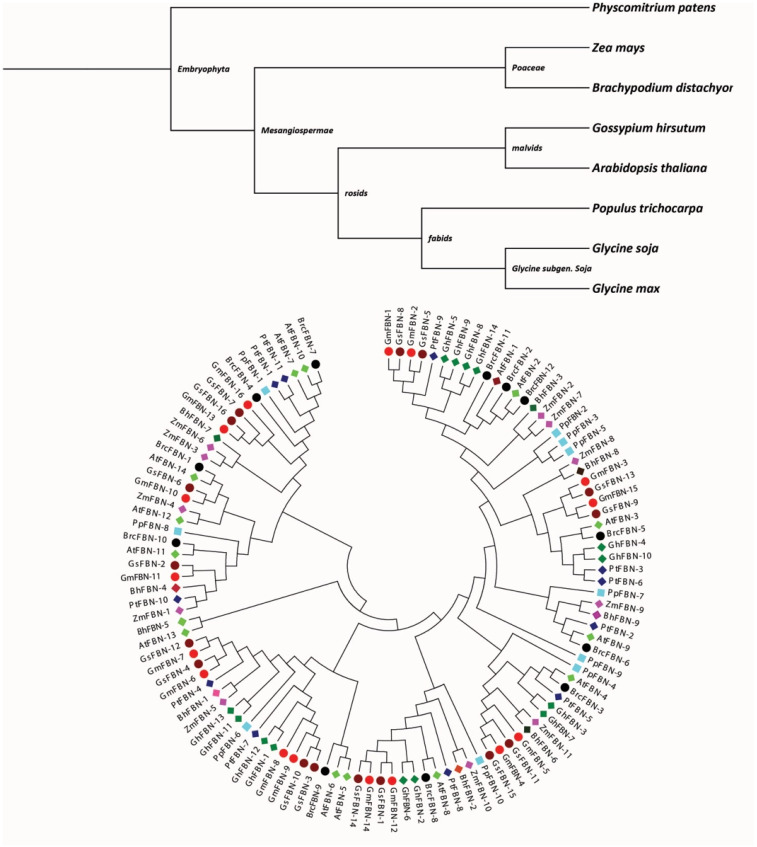
Phylogenetic analysis of FBNs proteins: The phylostartum analyses of FBN gene family; Phylogenetic analysis among *G. max*, *G. soja*, *B. distachyon*, *G. hirsutum*, *P. trichocarpa*, *P. patens*, *Z. mays*, *A. thaliana*, and *B. rapa* using the MEGA11 by the neighbor-joining method.

To understand the evolutionary relationships among FBNs and explore the extent of diversification of this gene family, a phylostartum and phylogenetic tree was constructed. Phylostratum analysis of FBN genes identified the primitive ancestry as FBN genes were present in *P. patens* (Bryophyte), one of the early plant pedigrees (Figure 5). The FBN gene family existed in *A. comosus* (angiosperm), *A. thaliana*, *G. max*, *G. soja*, *G. hirsutum*, *B. rapa* and *P. trichocarpa* (dicotyledons), *B. distachyon* and *Z. mays* (monocotyledons), and *P. patens* (Bryophyte) (Supplementary Table S2). According to these findings, FBNs that evolved from primitive plants and potential orthologous genes are present throughout kingdom Plantae. Next, an NJ tree was constructed to estimate the deeper relationships of FBN genes in the studied organism. Upon closer inspection of the phylogenetic tree, six distinct clades were identified, each representing a distinct stage in the transition from single-celled to multicellular life. Clade-1 was the largest and comprised 32 genes from virtually all species. Clade-1 contained genes from angiosperm, dicots, monocots and bryophytes, indicating the evolution of these FBN genes after the split of lycophytes, chlorophytes and bryophytes. Hence Clade-1 can be considered the earliest group of FBN genes, having FBN gene family members from all the studied organisms. The phylogenetic analyses indicated that *G. max* experienced gene family expansion because it had more FBN genes compared to the other studied organisms, except *G. soja*. Generally, GmFBNs and *GsFBNs* cluster together in the phylogenetic tree, which can be interpreted as evidence of a common pedigree.

**Figure 6:**
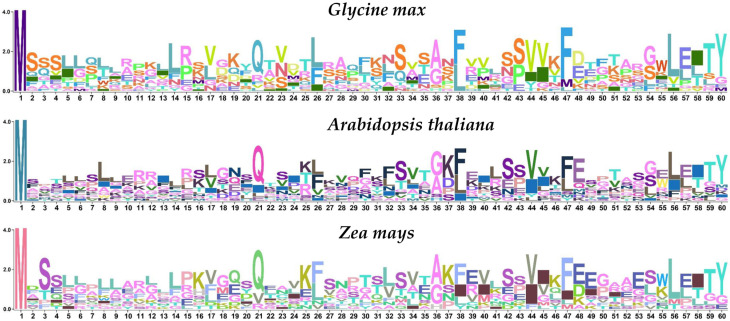
Conserved region (amino acid residues) sequence logo of *G. max*, *A. thaliana*, and *Z. mays*.

Overall, it was observed that the orthologs of the FBN gene family were present throughout the plant kingdom, indicating that the family originated from organisms such as *P. patens* through the course of evolution. In order to evaluate the extent of conservation within the FBN protein family, multiple sequence alignment was used to create sequence logos for each amino acid residue among *G. max*, *Z. mays*, and *A. thaliana*. We found that there were no conservation regions of *G. max* with *G. soja*, and *A. thaliana*, except M(1) in *G. max*, *Z. mays*, and *A. thaliana* (Figure 6). Additionally, sequence logos give a clear picture of how all the species are diverse. Furthermore, it can be deduced that the FBN gene family is universally less conserved among different species.

### 3.4. In-Silico Prediction and Physiochemical Properties of FBN in G. max

**Figure 7:**
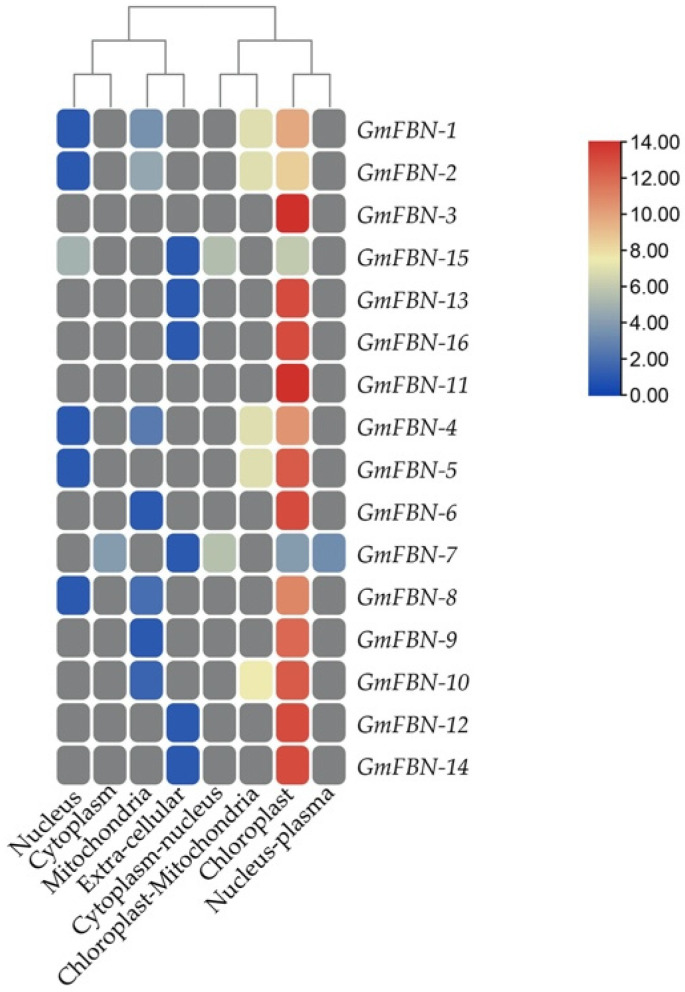
In-silico sub-cellular localization prediction of 16 GmFBNs.

As our main focus was *G. max* so further, basic information for GmFBNs, i.e., gene length, CDS length, protein length, protein molecular weight, isoelectric point, gravity, and predicted sub-cellular location are provided in Supplementary Table S3. GmFBN-1-GmFBN-16 included proteins with lengths ranging from 164 to 674 amino acids. The GmFBN genes’ CDS (coding sequence) lengths ranged from 495 bp to 2022 bp, and the gene lengths ranged from 1610 bp to 7747 bp, from GmFBN-1 to GmFBN-16. The calculated molecular weight of GmFBN-1–GmFBN-16 was between 17,669.25 and 75,594.61 kDa. The theoretical isoelectric point (pI) for GmFBN proteins was studied to be in the range of 5.24 to 9.69. As a further note, GmFBN proteins had GRAVY values ranging from –0.039 to –0.327 (Supplementary Table S3). GmFBN sub-cellular localizations were hypothesized to help identify its expression in distinct cellular and organelle contexts. The predicted sub-cellular localizations of GmFBN indicated that they were all found in the inner membrane. It was predicted that the vast majority of GmFBNs would be found in chloroplasts (Figure 7).

### 3.5. Chromosomal Distribution, Gene Duplication and Synteny Analysis

**Figure 8:**
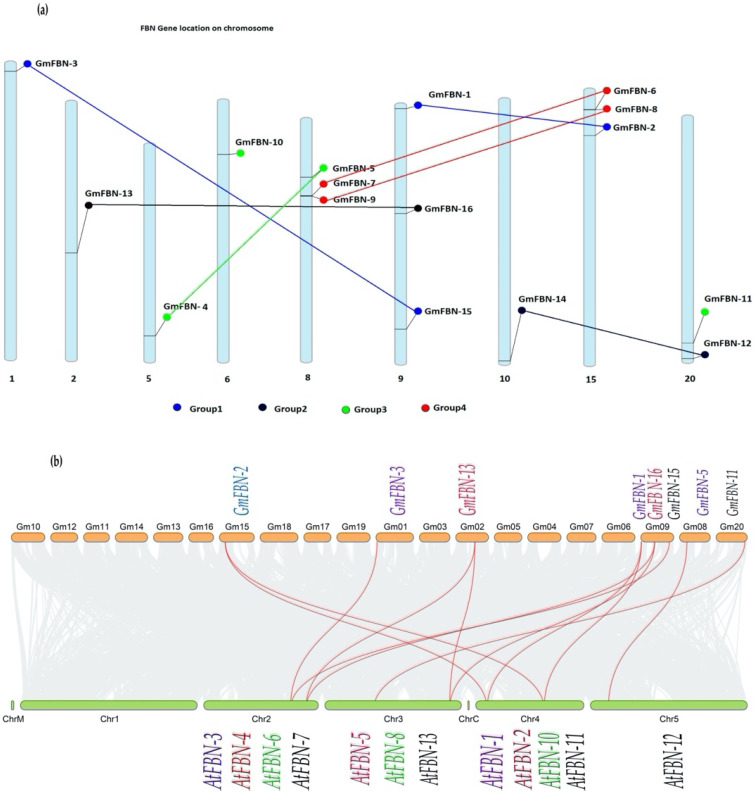
The chromosome location of GmFBNs genes and synteny analysis between *G. max* and *A. thaliana*: (**a**) Members of FBNs were divided into groups present on different chromosomes; blue indicates the first group, black indicates the second group, green indicates the third group, and red indicates the fourth group; (**b**) Synteny analysis for duplicated gene pairs in *G. max* and *A. thaliana*. Chromosomal lines are represented by red.

The position of the GmFBN genes on the *G. max* chromosomes was predicted through chromosomal localization analysis. A total of 16 GmFBN genes were distributed on the nine chromosomes. GmFBN-3, GmFBN-13, GmFBN-4, and GmFBN-10 were mapped on chromosomes 1, 2, 5, and 6 respectively. GmFBN-5, GmFBN-7, and GmFBN-9 were identified on chromosome 8. GmFBN-1, GmFBN-16, and GmFBN-15 were mapped on chromosome 9. GmFBN-14 was located on chromosome 10. GmFBN-2, GmFBN-6, and GmFBN-8 were located on chromosome 15 while GmFBN-11 and GmFBN-12 were located on chromosome 2. In addition, this analysis revealed there are seven paralogs (Supplementary Table S4). The segmental duplication event was detected between GmFBN-1 and GmFBN-2, GmFBN-3 and GmFBN-15, GmFBN-4 and GmFBN-5, GmFBN-6 and GmFBN-7, GmFBN-8 and GmFBN-9, GmFBN-13 and GmFBN-16, and GmFBN-12 and GmFBN-14, respectively (Figure 8a). We performed synteny analyses, in which we observed the location of two genes on a chromosome to see how they were linked. The synteny analysis proved that all GmFBNs had a certain degree of genetic similarity.

The expansion of a gene family is dependent on whole genome duplication, tandem duplication, and segmental duplication. Among the analyzed gene pairs, 10 were found to have been duplicated due to segmental duplication. GmFBN-1 represented collinearity between chromosome 9 in *G. max* and chromosome 4 in *A. thaliana*. The GmFBN-2 represented collinearity between chromosome 15 in *G. max* and chromosome 4 in *A. thaliana*. GmFBN-3 represented collinearity between chromosome 1 in *G. max* and chromosome 2 in *A. thaliana*. GmFBN-5 represented collinearity between chromosome 8 in *G. max* and chromosome 5 in *A. thaliana*. GmFBN-11 represented collinearity between chromosome 20 in *G. max* and chromosome 3 in *A. thaliana*. GmFBN-13 represented collinearity between chromosome 2 in *G. max*; its one copy was present on chromosome 2 and its second copy was present on chromosome 3 in *A. thaliana*. GmFBN-15 represented collinearity between chromosome 9 in *G. max* and chromosome 2 in *A. thaliana*. GmFBN-16 indicated collinearity between chromosome 2 in *G. max*; its one copy was present on chromosome 2 and its second copy was present on chromosome 3 in *A. thaliana*, respectively (Figure 8b).

The synteny analysis between *G. max* and *A. thaliana* revealed that both species had conserved regions. The results showed that there was strong conservation of some gene loci between GmFBN and AtFBN. According to Darwinian theory (natural selection), we investigated non-synonymous and synonymous divergence levels (Ka and Ks, respectively) between GmFBN gene pairs. It was established that six duplicated gene pairs showed Ka/Ks values <0.5, whereas one duplicated gene pair showed Ka/Ks values of 0.5 (Table 1 and Supplementary Table S4). While no pairs showed Ka/Ks value greater than 1. Ka/Ks of the examined gene pairs were typically less than 1, indicating that the *GmFBN* gene family was subject to purifying selection pressure with limited functional divergence. This functional divergence might take place after a segmental duplication event during polyploidization followed by hybridization in soybean evolution.

### 3.6. In-Silico Gene Expression Analysis GmFBN

**Figure 9:**
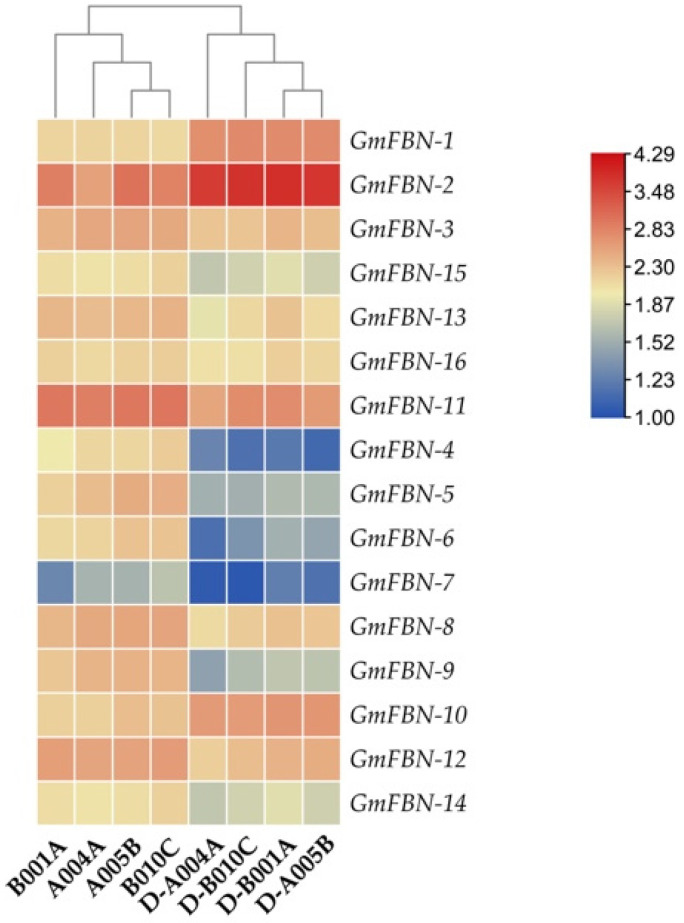
Expression analysis of GmFBNs genes. Expression levels from high (red) to medium (blue) to low (yellow) are shown. A004A, A005B, B001A, B010C = soybean plant grown under normal conditions; D-A004A, D-A005B, D-B001A, D-B010C = soybean plant grown under drought conditions [25].

The Gene Expression Omnibus (GEO) dataset was used to investigate *GmFBN* gene expression. As a transcriptional activator, *GmFBN* controls the activity of several genes involved in responding to stress. These genes are involved in the Ca^2+^ signaling route, the ABA signaling system, or both. Expression analyses through RNA-seq were performed on three different samples of transgenic soybeans (designated A004A, A005B, and B010C), and on null (control) plants for comparison [25]. Gene expression profiles in response to drought were generated using the sequence data as reported by Wei et. al. [25]. In response to drought, the soybean plant relied on *GmFBN-2*, *GmFBN-1*, *GmFBN-10*, *GmFBN-11*, and *GmFBN-15* members of the fibrillin gene family that were found to be strongly upregulated by the gene expression analyses (Figure 9).

### 3.7. Development of SNP Based Marker Using CAPS Technique and Marker Trait Association Analysis

**Figure 10:**
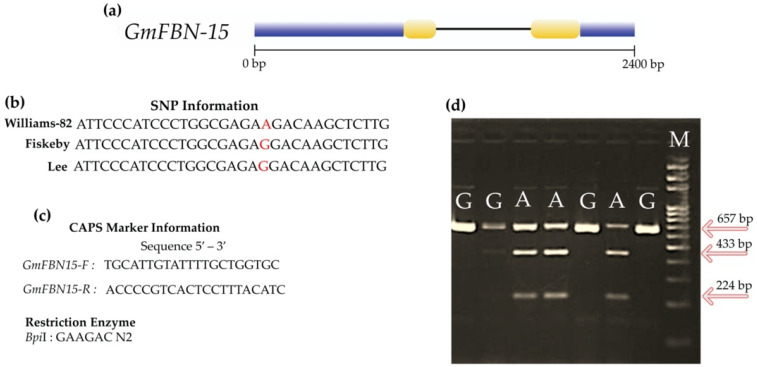
Sequence polymorphism and molecular marker development of *GmFBN-15*. (**a**) Showing *GmFBN-15* gene structure with intron, exons region, and untranslated regions; black line indicates intron, yellow box indicates exon, and blue box indicates untranslated regions. (**b**) Presents the polymorphic site in three different soybean accessions. (**c**) Primers for CAPS marker and restriction enzyme information. (**d**) Agarose gel electrophoretic profile of selected samples showing the banding patterns run with M = 100 bp DNA ladder. Undigested PCR amplicon size is 657 bp. *Bpi*I digested the amplified product into two fragments i.e., 433 bp and 224 bp.

**Figure 11:**
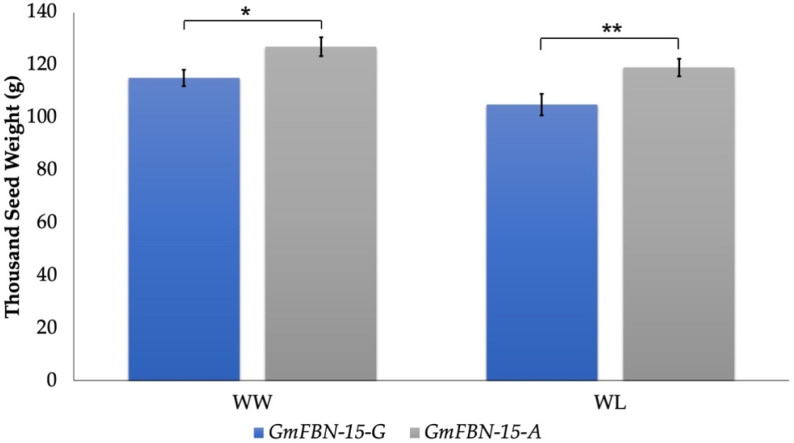
Genotyping results of soybean accessions carrying *A*-allele or *G*-allele under well-water (WW) and water-limited (WL) conditions; Error bar denotes standard error; * *p* < 0.05, ** *p* < 0.01.

For *GmFBN-15*, a polymorphic site was identified in the coding region. To distinguish the allelic variation, one molecular marker (GmFBN-15-F/R) was developed using the CAPS technique. One allelic variation (*A*-allele) had a *Bpi*I restriction site, un-restricted for *G*-allele (Figure 10). For *GmFBN-15*, 30% of the studied germplasm possessed a *GmFBN-15*-*A* allele, while 70% possessed a *GmFBN-15-G* allele. Association analysis was performed on studied traits under both growing environments, and a statistically non-significant association was found for all traits except thousand seed weight. *GmFBN-15-A* showed an association with higher thousand seed weight under both water regime conditions (Figure 11).

## 4. Discussion

*FBN* genes have been characterized in different organisms, including Arabidopsis, brassica, rice, cucumber, wheat, chickpea, and tobacco [4,7,8,9,10,13]. To date, no comprehensive assessment of *GmFBN* genes has been carried out. We performed detailed bioinformatics analyses of *GmFBN* in order to characterize *FBN* and its potential use in soybean molecular breeding. Overall, the results of this work provide basic information and lay the foundation for further work into the *FBN* gene function in soybean. In this study, we identified 16 *FBN* genes in the soybean genome. These genes were distributed on nine chromosomes (Figure 8a). All of the identified *GmFBN* genes were localized in chloroplast and contained chloroplast transit peptides. These results provided clear evidence that many *FBNs* might play a role in photosynthesis. GRAVY was used to determine the hydrophobicity of each protein sequence, with higher positive values suggesting a more hydrophobic protein. Protein hydrophobicity may be influenced by their spatial organization and the proportion of hydrophobic residues they contain [10].

To analyze the evolutionary relationships of the *FBN* genes, we constructed a phylogenetic tree with a total of 113 *FBN* genes in nine investigated species, including *A*. *comosus* (angiosperm), *A. thaliana*, *G. max*, *G. soja*, *G. hirsutum*, *B. rapa* and *P. trichocarpa* (dicotyledons), *B. distachyon* and *Z. mays* (monocotyledons), and *P. patens* (Bryophyte). Members of the *GmFBNs* gene family were distributed widely in all the phylogenetic groups. Based on these findings, it appears that the *FBN* genes in these monocots that were in the same clade might have a similar biological function. Furthermore, the evolutionary study provides a base for future functional studies on *FBN* genes in soybean.

We identified 16 *GmFBN* genes in the *G. max* genome (~978 Mb) which were higher in number compared to *P. trichocarpa* (~500 Mb genome), *P. patens* (~500 Mb genome), and *A. thaliana* (~125 Mb genome). The probable reason for the larger number of *FBNs* in *G. max* (16), as compared to *P. trichocarpa* (11), *P. patens* (10), and *A. thaliana* (14), is that *G. max* is allotetraploid and the uneven distribution of *GmFBNs* on *G. max* chromosomes shows probable gene addition through segmental duplication incidents. It has been reported that gene duplication and divergence generally leads towards evolution [1,2,21]. The gene duplication phenomenon is crucial for speciation and adaptability in ever-changing environmental conditions. We found duplicated genes present on different chromosomes of the same sub-genome. This duplication might be a consequence of segmental or whole genome duplication (Figure 8b). Almost 65 MYA segmental and whole genome duplications in primitive plants have contributed to the expansion of the number of gene families. Hence it can be anticipated that *FBN* also expanded and contributed to the genome complexity of plants.

Gene structure analyses exhibited that *GmFBNs* have varied intron lengths that might play crucial roles in the functional divergence of *GmFBNs.* It has been well documented that intron played an important role in the evolution of different species [1,2].

Gene function determines the extent to which a gene is expressed in various tissues and at various times during development. Several research works have reported the effects of abiotic stress on *FBNs*, and it is evident that they are regulated by a wide range of biological and environmental factors [10,11,12,25]. The expression of *cis*-regulatory elements in downstream genes is regulated by transcription factors, which play a role in a wide range of biological activities [25]. The promoter sequences of the *GmFBN* genes contained numerous *cis*-regulatory elements. Hormonal-responsive elements, including MeJA, abscisic acid, gibberellic acid, salicylic acid, and auxin, were also present in this blend, and these factors are connected in their response to light and drought. Unexpectedly, several components associated with light sensitivity were also present in all *GmFBN* genes.

Over the last ~100 years soybean genetic gain is estimated to be ~12 kg/acre [26]. This improvement has largely been attained by breeding for grain yield following conventional breeding and selection approaches. The coupling of these approaches with genomic tools will definitively fast-track the genetic gain in crop plants [27]. The soybean PAN-genome has the potential to close the phenotype-genotype gap in soybean breeding. PAN-genome has recently been utilized to identify genes in *G. soja* that regulate flowering timing [3]. In order to keep improving soybeans, it is essential to use marker-assisted selection to identify important best genes. If efficient molecular platforms are available, the application of elite allelic variants in cultivars can be increased [1,2,27,28,29,30]. The sequence polymorphism of *GmFBN* was investigated in this study using the *G. max* PAN-genome. All *GmFBN* genes were analyzed for sequence polymorphism; however, allelic variation was only found in the *GmFBN-15* coding area. Therefore, in order to conduct marker-trait association studies, only this candidate gene was considered. Allele fixation throughout evolution may account for the lack of variation in the other *GmFBN* genes, or this may be related to the small sample size of accessible *G. max* accessions in PAN-genomics research. Additional research is needed to confirm or explore these two possibilities. SNP-based molecular markers are easy to deal with and offer higher throughput compared to other markers [31,32]. A single nucleotide polymorphism was found in *GmFBN-15*. Williams-82 contained A-allele while the other two cultivars contained G-allele at that point [Figure 11b]. Although there was only one restriction site in 657 bp fragment length and it should produce only two bands (at 433 bp and 224 bp) on agarose gel, it still produced three bands for the genotypes carrying A-allele. The probable reason is that the GmFBN-15-F/GmFBN-15-R (primer pair) might have amplified the paralog of *GmFBN-15*, which is *GmFBN-3* present on chromosome 1.

This study provides useful information for *GmFBN* genes in that members of the *FBN* gene family may improve plant performance for abiotic stress and increase yield-related traits in different stages, and can be used further for soybean breeding programs. The findings presented here will be useful for future studies into the biological roles of *GmFBN* genes.

## 5. Conclusions

In this study, we analyzed 113 different *FBN* genes in nine different organisms. Bioinformatics analyses revealed that *FBN* genes are relatively less conserved and contain *cis*-acting elements necessary for plant growth and development, and respond to various external stimuli, including drought. Moreover, marker-trait association analyses also highlighted the potential role of *FBN* in soybean molecular breeding.

## Figures and Tables

**Table 1:** Calculation of nonsynonymous (Ka) and synonymous (Ks) substitution rates and determination time (MYA) of soybean *FBN* genes.

Gene 1 ID	Gene 2 ID	*Ka*/*Ks*	Time (MYA)
*GmFBN-1*	*GmFBN-2*	0.129409128	1
*GmFBN-3*	*GmFBN-15*	0.234590794	2
*GmFBN-12*	*GmFBN-14*	0.172072626	1
*GmFBN-13*	*GmFBN-16*	0.13874396	1
*GmFBN-4*	*GmFBN-5*	0.504124568	6
*GmFBN-10*	*GmFBN-7*	0.482073521	3
*GmFBN-8*	*GmFBN-9*	0.437348305	3

## Data Availability

The datasets presented in this study can be found in online repositories. The names of the repository/repositories and accession number(s) can be found in the article Supplementary Material.

## Supplementary Materials

The following supporting information can be downloaded at: https://www.mdpi.com/article/10.3390/genes14061188/s1. Table S1: Weather conditions; Table S2: Gene IDs; Table S3: Physio-chemical properties; Table S4: Collinearity, synteny and Ka/Ks analyses of *GmFBN* gene family.
